# Diosgenin Reduces Acute Kidney Injury and Ameliorates the Progression to Chronic Kidney Disease by Modifying the NOX4/p65 Signaling Pathways

**DOI:** 10.1021/acs.jafc.4c04183

**Published:** 2024-07-29

**Authors:** Chih-Hung Chiang, Tien-Yun Lan, Jung-Hung Hsieh, Su-Chu Lin, Jaw-Wen Chen, Ting-Ting Chang

**Affiliations:** † Division of Urology, Department of Surgery and Department of Research and Development, Taoyuan General Hospital, Ministry of Health and Welfare, Taoyuan 330, Taiwan; ‡ Department of Urology, National Taiwan University Hospital, Taipei 100, Taiwan; § Department and Institute of Pharmacology, School of Medicine, National Yang Ming Chiao Tung University, Taipei 112, Taiwan; ∥ Department of Surgery, Taipei Veterans General Hospital, Yuan-Shan Branch, Yilan 264, Taiwan; ⊥ Department of Medical Research and Education, Taipei Veterans General Hospital, Yuan-Shan Branch, Yilan 264, Taiwan; # Cardiovascular Research Center, Taipei Medical University Hospital and Taipei Medical University, Taipei 110, Taiwan; ∇ Division of Cardiology, Department of Medicine and Department of Research, Taipei Medical University Hospital, Taipei 110, Taiwan; ○ Division of Cardiology, Department of Medicine, Taipei Veterans General Hospital, Taipei 112, Taiwan; ◆ Cardiovascular Research Center, National Yang Ming Chiao Tung University, Taipei 112, Taiwan; ¶ Biomedical Industry Ph.D. Program, National Yang Ming Chiao Tung University, Taipei 112, Taiwan

**Keywords:** acute kidney injury, chronic kidney disease, diosgenin, fibrosis, inflammation

## Abstract

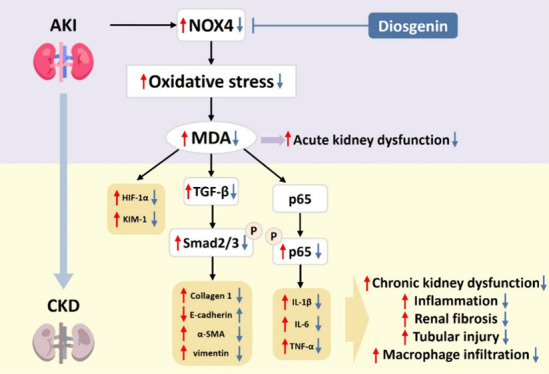

Acute kidney injury (AKI), if not well controlled, may progress to chronic kidney disease (CKD). Diosgenin is a natural phytosteroid sapogenin from plants. This study aimed to investigate the mechanistic effects of diosgenin on AKI and AKI related development of CKD. The mouse model of ischemia/reperfusion (I/R)-induced AKI was used, and its progressive changes were followed. Human renal proximal tubular epithelial cells were used, and hypoxia stimulation was applied to mimic the in vivo I/R. Diosgenin, given after renal injury, preserved kidney function, as evidenced by a reduction in serum levels of BUN, creatinine, and UACR in both acute and chronic phases of AKI. Diosgenin alleviated I/R-induced tubular injury and prevented macrophage infiltration and renal fibrosis in AKI mice. Furthermore, diosgenin also mitigated the development of CKD from AKI with reduced renal expression of inflammatory, fibrotic, and epithelial–mesenchymal transition markers. In human renal tubular epithelial cells, diosgenin downregulated the hypoxia-induced oxidative stress and cellular damages that were dependent on the NOX4/p65 signaling pathways. Taken together, diosgenin treatment reduced I/R-induced AKI and ameliorated the progression to CKD from AKI probably by modifying the NOX4/p65 signaling pathways.

## Introduction

Acute kidney injury (AKI) is defined as a sudden loss of kidney function with a rapidly rising serum creatinine level or decreased urine output. AKI occurs in about 10–15% of patients in the hospital, especially in the intensive care unit, where almost 50% of patients suffer from AKI.^1^ AKI is highly associated with morbidity, which causes about 1.7 million deaths per year, but currently lacks effective pharmacological treatments.^2^ Given the high morbidity in AKI patients, the early intervention and long-term follow-up after AKI are clinically critical. AKI is considered to be closely related to chronic kidney disease (CKD). The risk of AKI patients developing CKD is increased even if their kidney function is fully recovered.^3^ Thus, AKI is a tough global health concern, and proper interventions are urgently needed.

There are several potential causes of AKI, including renal hypoperfusion, nephrotoxin exposure, sepsis, etc.^1,4,5^ One of the leading causes of AKI is ischemia/reperfusion (I/R) injury.^6^ The kidney receives about 25% of the cardiac output; therefore, any failure of the systemic circulation or blocking of intrarenal circulation could negatively affect renal perfusion.^7^ With decreasing renal perfusion or ischemia, there is not enough ATP to maintain essential processes, leading to the massive generation of reactive oxidative species (ROS) during reperfusion in tubular epithelial cells, which may result in cell apoptosis.^8^ The formation of ROS also promotes inflammation and injury in the kidney.^9^ ROS activate inflammatory cytokines, which accumulate in the renal tubules, and eventually cause the death of tubular epithelial cells.^10^ NADPH oxidase (NOX) 4 is highly expressed in the kidney and was shown to play a critical role in I/R injury-induced ROS generation in renal tubular epithelial cells.^11,12^ Nuclear factor kappa B (NF-κB), which is sensitive to redox reactions, is significantly elevated after I/R in the kidney, leading to the transcription of inflammatory proteins, particularly tumor necrosis factor-α (TNF-α) and interleukin (IL)-6.^13,14^

Kidney inflammation, fibrosis, and tubular epithelial cell damage were reported to participate in the progression of AKI to CKD.^15^ Kidney fibrosis is an adaptive mechanism for the repair of renal tissues in the short term, but continuous fibrosis can lead to long-term organ failure.^16^ I/R injury-induced ROS formation from epithelial cells can activate the transforming growth factor-β (TGF-β)/Smad 2/3 pathway and cause collagen 1 deposition and α-smooth muscle actin (SMA) accumulation, which is the critical process in renal fibrogenesis in AKI-to-CKD progression.^17,18^ Furthermore, the epithelial-mesenchymal transition (EMT) occurs in the epithelial cells of injured kidney tubules. The epithelial cells, through a phenotype change, transform into fibroblasts. EMT is considered a key mechanism of kidney fibrogenesis.^16,19^ The activation of ROS induces the NF-κB signaling pathway, which plays a central role in EMT progression by upregulating the expression of EMT-related proteins, such as E-cadherin and vimentin.^20,21^

Diosgenin is a natural phytosteroid sapogenin that is derived from a variety of plants, such as wild yam (*Dioscorea villosa*) and fenugreek seed (*Trigonella foenum-graecum* L.). Diosgenin presented a protective effect against kidney injury in a 3-chloro-1,2-propanediol (3-MCPD)-induced kidney injury model.^22^ Diosgenin could also ameliorate diabetic kidney damage and suppress renal NOX4 expression in diabetic nephropathy rats. In human kidney proximal tubule epithelial cells, diosgenin could inhibit the generation of ROS and reduce the expression of apoptotic proteins, such as Apaf-1, CytC, cleaved caspase 3, and cleaved caspase 9 under high-glucose conditions.^23^ However, the potential effects of diosgenin on I/R injury-induced AKI as well as the development of CKD after AKI have not been well explored. In the current study, we hypothesized that diosgenin may reduce the damages of kidneys from I/R-induced AKI and prevent the progression of AKI to CKD by modifying NOX4/p65 signaling pathways. This study was therefore conducted to explore the effects of diosgenin on AKI and the consequent development of CKD after AKI in vivo and the mechanistic effects of diosgenin in hypoxia-stimulated renal proximal tubular epithelial cells in vitro (that is, mimic the I/R injury in vivo). The findings of this study may provide an important theoretical basis to using diosgenin as a novel renal protection strategy for ischemic related AKI and the consequent development of CKD.

## Materials and Methods

### Animal Model of AKI

Six week-old male C57BL/6 mice were purchased from the National Laboratory Animal Center (Taipei, Taiwan). The mice were housed in a 12 h light/dark cycle in specific, pathogen-free conditions. After acclimating for 2 weeks, unilateral renal I/R injury surgery was performed. In detail, the right renal pedicle was identified, and a right nephrectomy was performed. The left renal pedicle was then identified and clamped by using a small nontraumatic clamp. After visual confirmation of ischemic changes, the kidney was placed back into the peritoneal cavity, and vessel occlusion was maintained for 45 min. The clamp was then released, and the blood flow was visually confirmed to be restored. After the induction of AKI, diosgenin was given once a day by tube feeding at doses of 1 or 10 mg/kg/day for 21 days. The mouse model of AKI and doses of diosgenin used in the in vivo study were selected based on references.^23,24^ The animals were raised according to the regulations of the Animal Care Committee of National Yang Ming Chiao Tung University. All animal experiments were approved by the Institutional Animal Care and Use Committee (IACUC) of the National Yang Ming Chiao Tung University (IACUC No. 1110907).

### Blood and Urine Biochemistry

Given that the uptake of diet may affect serum levels of BUN and creatinine,^25^ blood samples were harvested from the submandibular vein of the mice after 6 h of fasting. Blood samples were clotted for 30 min at room temperature and centrifuged at 3,000*g* for 15 min at 4 °C to collect the serum. To evaluate the serum blood urea nitrogen (BUN) and creatinine levels, 10 μL of serum was added to a FUJI DRI-CHEM SLIDE (Fuji; BUN-PIII and CRE-P III) and detected with a DRICHEM NX500i chemistry analyzer. Serum malondialdehyde (MDA) levels were measured by MDA Assay kits (Abcam, ab118970, Cambridge, UK) based on the manufacturer’s instructions.

Mice were placed in metabolic cages to collect urine for 24 h. Urine samples were collected 3 days and 3 weeks after the I/R injury. Albuminuria was quantitated by the urine albumin:creatinine ratio (UACR). Urine albumin and creatinine levels were detected using ELISA kits (Exocell, #1011 and #1012, Township, NJ, USA) based on the manufacturer’s instructions.

### Histological Analysis

Samples were fixed in 10% paraformaldehyde and dehydrated. After being embedded in paraffin, samples were cut into 3 μm-thick sections and stained with hematoxylin and eosin (H&E), periodic acid–Schiff (PAS) stain, and also Masson’s trichrome stain. Tubulointerstitial injury in PAS-stained sections was classified as tubular dilation with epithelial and tubular atrophy. Interstitial fibrosis in Masson’s trichrome-stained sections was defined as areas that appeared dark blue in color due to accumulation of extracellular matrix. Six mice per group were randomly selected, their kidney sections were examined at ×100 magnification, and 10 nonoverlapping regions were selected from the entire cortical and outer medulla areas. The extent of tubulointerstitial injury and interstitial fibrosis were evaluated as a ratio relative to the entire cortical and outer medulla areas. In addition, samples were stained with F4/80 (Novus, NB600–404, Littleton, CO, USA) to evaluate macrophage infiltration. The ratio of F4/80-positive area relative to total kidney cortex area was calculated by using ImageJ software.

### Cell Culture and In Vitro Hypoxia Model Mimic I/R

Human kidney proximal tubule epithelial HK-2 cells were purchased from the Bioresource Collection and Research Center (60097, Hsinchu, Taiwan). The cells were cultured in keratinocyte serum-free medium (K-SFM) (Gibco, 17,005–042, Waltham, MA, USA) in a humidified atmosphere with 5% CO_2_ at 37 °C. HK-2 cells (8 × 10^4^ cells per well) were seeded into a 6-well plate, grown for 2 days, and starved in serum-free Dulbecco’s modified Eagle medium (DMEM) for a day before performing the hypoxia injury.

To mimic ischemia, the HK-2 cells were incubated in the hypoxia conditions in Hank’s Balanced Salt Solution (HBSS; Gibco, 14,185,052, Waltham, MA, USA) in a humidified atmosphere with 1% O_2_, 94% N_2_, and 5% CO_2_ at 37 °C for 6 h. To mimic reperfusion, the HK-2 cells were incubated in K-SFM and treated with diosgenin at 0.1 and 10 μM under a humidified atmosphere with 21% O_2_, 74% N_2_, and 5% CO_2_ at 37 °C for an hour. The doses of diosgenin used in the in vitro study were selected according to ref (26). The control group cells were subjected to the same treatments as the hypoxia group but were not exposed to hypoxia conditions.

### Cell Viability Assay

HK-2 cells (2 × 10^4^ cells per well) were seeded into 24-well plates, grown for 2 days, and starved in serum-free DMEM for a day. The cells were subjected to hypoxia injury protocol described above before the cell viabilities were measured by the cell counting kit-8 (Dojindo Molecular Technologies, Inc., Rockville, MD, USA) assay based on the manufacturer’s instructions. After reperfusion, the cells were incubated in 250 μL of K-SFM with 25 μL of the cell counting kit-8 solution under a humidified atmosphere with 21% O_2_, 74% N_2_, and 5% CO_2_ at 37 °C for an hour and the absorbance at 450 nm was measured.

### ROS Generation Assay

The production of hydrogen peroxide (H_2_O_2_) was detected as a quantitative measure of ROS generation by using the Amplex Red Hydrogen Peroxide/Peroxidase Assay Kit (Invitrogen, #A22188, Carlsbad, CA, USA) based on the manufacturer’s instructions. The Amplex Red reagent combined with horseradish peroxidase is used to detect H_2_O_2_ released from cells. For this assay, HK-2 cells were seeded at a density of 2 × 10^4^ cells per well in 24-well plates, grown for 2 days, and starved in serum-free DMEM for a day. To mimic ischemia, the HK-2 cells were incubated in HBSS under hypoxic conditions at 37 °C for 6 h. To mimic reperfusion, the HK-2 cells were incubated in K-SFM and treated with diosgenin at 0.1 and 10 μM under normoxia conditions for 1 h. The control group was subjected to the same treatment but was not exposed to hypoxia. After hypoxia, 25 μL of the medium was collected and mixed with 25 μL of the reaction buffer and 50 μL of the working solution and then incubated in a dark environment for 30 min. After incubation, the fluorescence was determined by using a microplate reader with an excitation wavelength of 530 nm and emission wavelength of 590 nm.

### TUNEL Assay

The one-step TUNEL Assay Kit (Elabscience, E-CK-A320, Houston, TX, USA) was used to detect the level of cell apoptosis based on the manufacturer’s instructions. The DNA of apoptotic cells is cleaved into fragments, and the exposed 3′-OH of the broken DNA can be catalyzed by the Terminal Deoxynucleotidyl Transferase in the kit to produce fluorescence, which can be detected with a fluorescence microscope. HK-2 cells (4 × 10^4^ cells per well) were seeded into 12-well plates, grown for 2 days, and starved in serum-free DMEM for a day. Then, the HK-2 cells were incubated in HBSS under hypoxia conditions at 37 °C for 6 h and reperfusion conditions for an hour. The cells were fixed in 4% formaldehyde for 30 min at room temperature, and permeabilized by Triton X-100 (0.1%) at 37 °C. After incubation, the cells were treated with labeling working solution for 30 min at 37 °C. Cellular DNA fragments were stained with FITC, and the cell nuclei were stained with DAPI. Fluorescence images were taken using a fluorescence microscope.

### Western Blotting

Equal amounts of protein samples were subjected to SDS-PAGE on 8–12% gradient gels and transferred to PVDF membranes. The membranes were incubated with antibodies against NOX4 (Thermo Fisher Scientific, PA5–88106, Waltham, USA), hypoxia-inducible factor (HIF)-1α (Cell Signaling Technology, #48085S, Danvers, MA, USA), kidney injury molecule-1 (KIM-1; R&D Systems, AF1817, Minneapolis, MN, USA), TGF-β (Cell Signaling Technology, #3711S, Danvers, MA, USA), p-Smad2/3 (Cell Signaling Technology, #8828S, Danvers, MA, USA), Smad2/3 (Cell Signaling Technology, #3102S, Danvers, MA, USA), collagen 1 (Cell Signaling Technology, #91144S, Danvers, MA, USA), NF-κB subunit p65 (BD, 0079008, East Rutherford, NJ), p-p65 (Cell Signaling Technology, #3031S, Danvers, MA, USA), IL-1β (Santa Cruz, sc-7884, Dallas, TX, USA), E-cadherin (Cell Signaling Technology, #3195S, Danvers, MA, USA), vimentin (Cell Signaling Technology, #5714S, Danvers, MA, USA), α-SMA (Cell Signaling Technology, #19245S, Danvers, MA, USA), IL-6 (Cell Signaling Technology, #12153S, Danvers, MA, USA), TNF-α (Cell Signaling Technology, #3037S, Danvers, MA, USA), caspase 3 (Cell Signaling Technology, #9662S, Danvers, MA, USA), and poly-ADP-ribose polymerase (PARP; Cell Signaling Technology, #9532S, Danvers, MA, USA). The immunoblot expression of NOX4, HIF-1α, KIM-1, TGF-β, Smad2/3, p-Smad2/3, collagen 1, p65, p-p65, E-cadherin, vimentin, α-SMA, IL-1β, IL-6, TNF-α, caspase 3, and PARP was normalized to β-actin expression measured using anti-β-actin (Santa Cruz, sc-81178, Dallas, TX, USA). For the analysis of kidney samples in vivo, four mice per group in each condition were analyzed for the quantification of proteins.

### Transfection of NOX4 siRNA

HK2 cells were transfected with the siRNA of NOX4 (Santa Cruz, sc-41586, Dallas, TX, USA) by using an oligofectamine (Thermo Fisher Scientific, 12252011, Waltham, USA) reagent based on the manufacturer’s instructions.

### Statistical Analysis

The results are presented as the mean ± standard deviation. Statistical analyses were performed using one-way ANOVA followed by post hoc Tukey’s multiple comparisons test. The results were considered significant at a *p* value of <0.05. The analyses were performed by using SPSS version 29.0 (Chicago, IL, USA).

## Results

### Diosgenin Preserved Renal Function in Acute and Chronic Phases of AKI Mice

**Figure 1:**
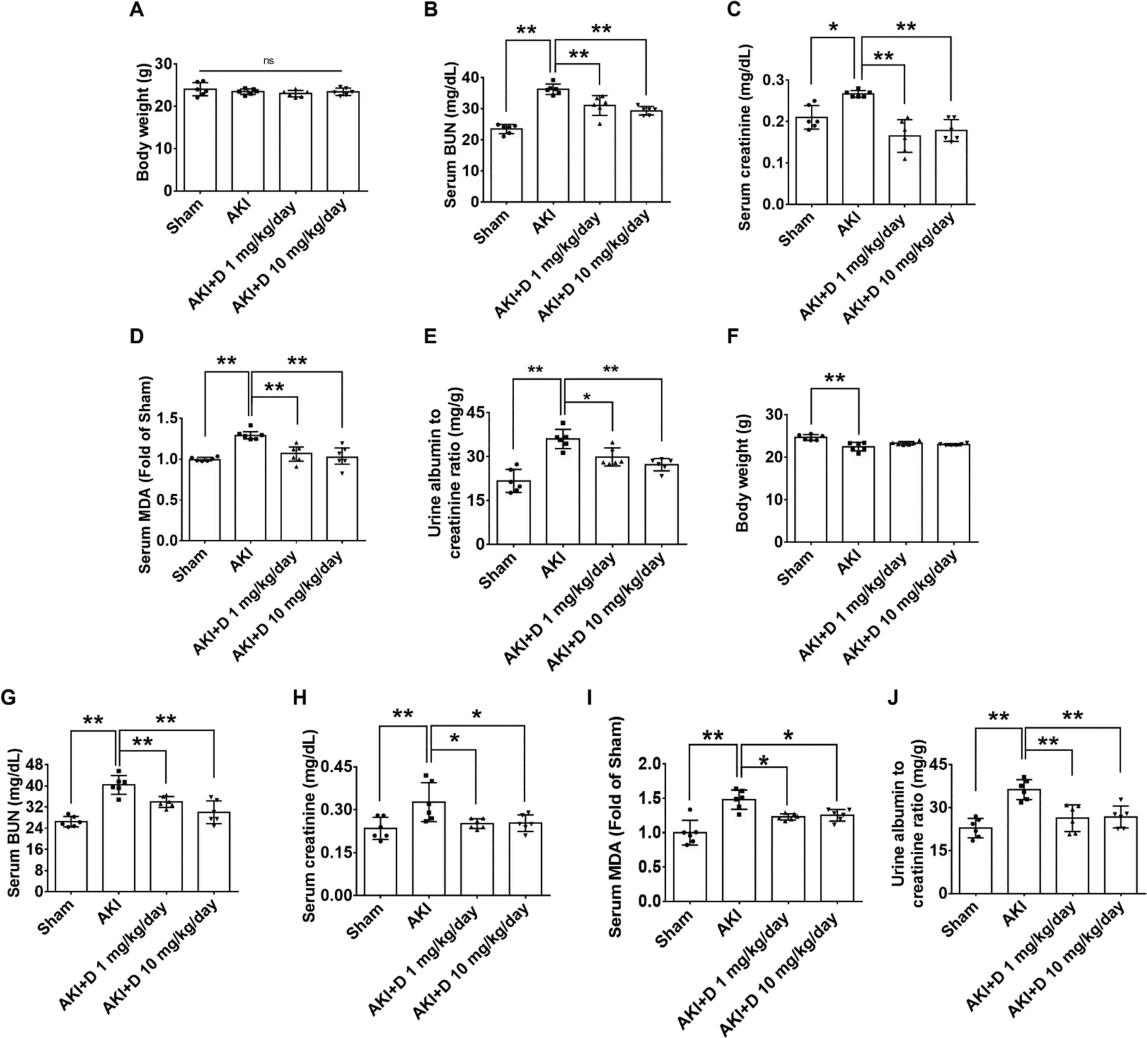
Diosgenin treatment protected the kidney from I/R-induced renal dysfunction in both acute and chronic phases of AKI. The body weight in each group of mice was measured after 3 days of AKI (*n* = 6; A). Serum BUN and creatinine levels were measured after 3 days of AKI (*n* = 6; B, C). Serum MDA levels were measured after 3 days of AKI (*n* = 6; D). The urine albumin–creatinine ratio was measured after 3 days of AKI (*n* = 6; E). The body weight in each group of mice was measured after 3 weeks of AKI (*n* = 6; F). Serum BUN, creatinine, and MDA levels were measured after 3 weeks of AKI (*n* = 6; G–I). The urine albumin–creatinine ratio was measured after 3 weeks of AKI (*n* = 6; J). AKI, acute kidney injury; BUN, blood urea nitrogen; D, diosgenin; MDA, malondialdehyde. *P < 0.05, **P < 0.01.

The body weight was not affected in AKI mice after 3 days of AKI (Figure 1A). The levels of serum BUN and creatinine were higher in AKI mice but decreased after diosgenin treatments 3 days post AKI (Figure 1B,C). The AKI mice showed elevated serum MDA levels, which were reduced in mice treated with diosgenin (Figure 1D). The AKI mice had a higher UACR, which was lowered by diosgenin treatment at 3 days post AKI (Figure 1E). The body weight of the AKI mice was decreased after 3 weeks of AKI (Figure 1F). Serum BUN, creatinine, and MDA levels as well as the UACR were higher in the AKI mice but decreased in the diosgenin-treated mice at 3 weeks post AKI (Figure 1G–J). These results showed the renoprotective effect of diosgenin in the acute and chronic phases of AKI.

### Diosgenin Attenuated Renal Damage of AKI as well as the Progression to CKD by Reducing Renal Tubular Injury, Fibrosis, and Macrophage Infiltration

**Figure 2:**
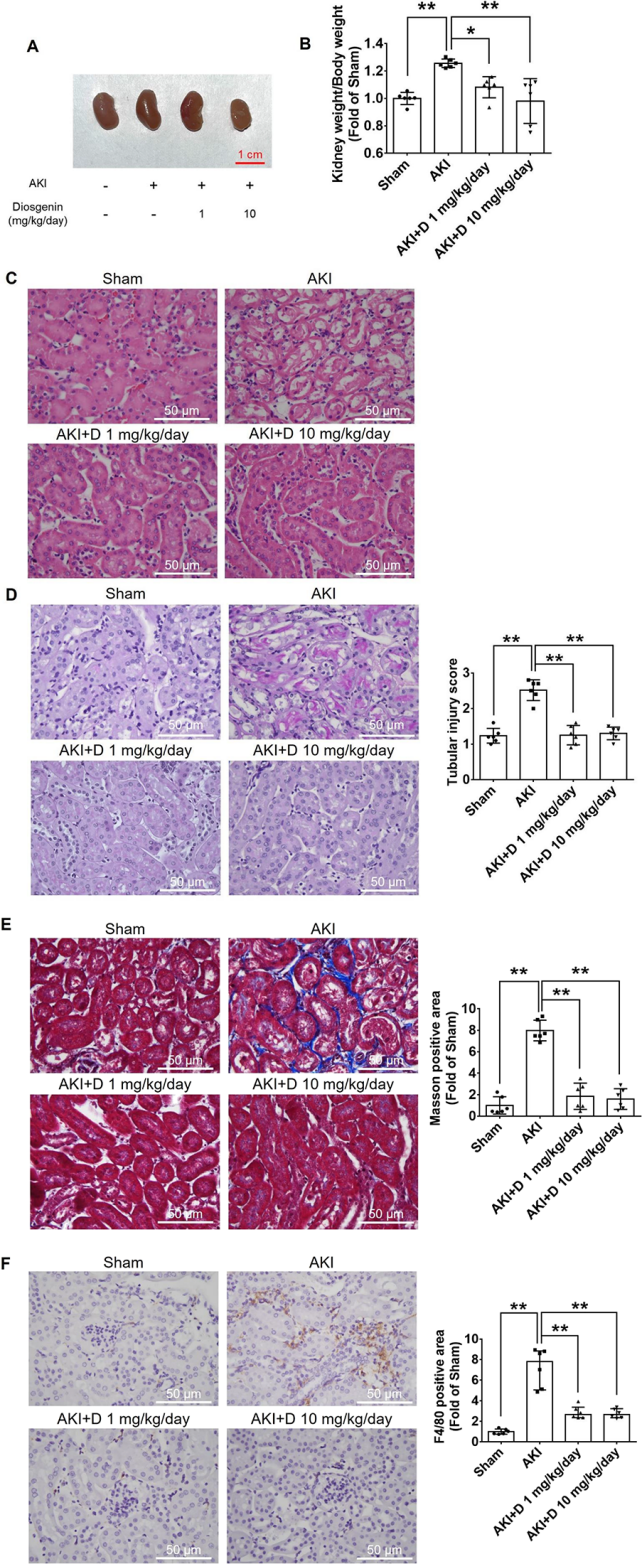
Diosgenin treatment attenuated tubular injury, fibrosis, and macrophage infiltration in the kidney tissues of AKI mice. Morphology of the kidney after 3 weeks of AKI (A). The kidney weight to body weight ratio was measured after 3 weeks of AKI (*n* = 6; B). Representative H&E staining of kidney sections (C). Representative PAS staining in tubulointerstitial lesions of kidney sections (*n* = 6; D). Representative Masson’s trichrome staining and fibrosis scores of kidney sections (*n* = 6; D). Representative Masson’s trichrome staining of a kidney section. Quantitative analysis of collagen deposition in the kidney at 3 weeks after AKI (*n* = 6; E). Representative F4/80 staining of a kidney section and quantitative analysis of F4/80-positive areas at 3 weeks after AKI (*n* = 6; F). AKI, acute kidney injury; D, diosgenin. *P < 0.05, **P < 0.01.

Diosgenin was found to attenuate kidney hypertrophy caused by AKI (Figure 2A). The kidney/body weight ratios of the AKI mice were elevated compared to the sham group but decreased following diosgenin treatments 3 weeks post AKI (Figure 2B). The diosgenin treatment alleviated the AKI-induced tubular injury by decreasing the size of lesions that formed due to death cell and tubular dilation in the kidney section (Figure 2C,D). Furthermore, the diosgenin treatment for 3 weeks led to a reduction in renal collagen deposition caused by AKI (Figure 2E). Additionally, the level of macrophage infiltration was heightened in AKI mice but decreased after diosgenin treatment at 3 weeks post-AKI (Figure 2F). These findings indicate that diosgenin effectively protected the kidney from tubular injury caused by AKI and mitigated the transition from AKI to CKD through suppression of the inflammatory responses and consequent kidney fibrosis.

### Diosgenin Reduced the Renal Inflammation, Fibrosis, and EMT Signaling Activity in the Chronic Phase of AKI in Mice

**Figure 3:**
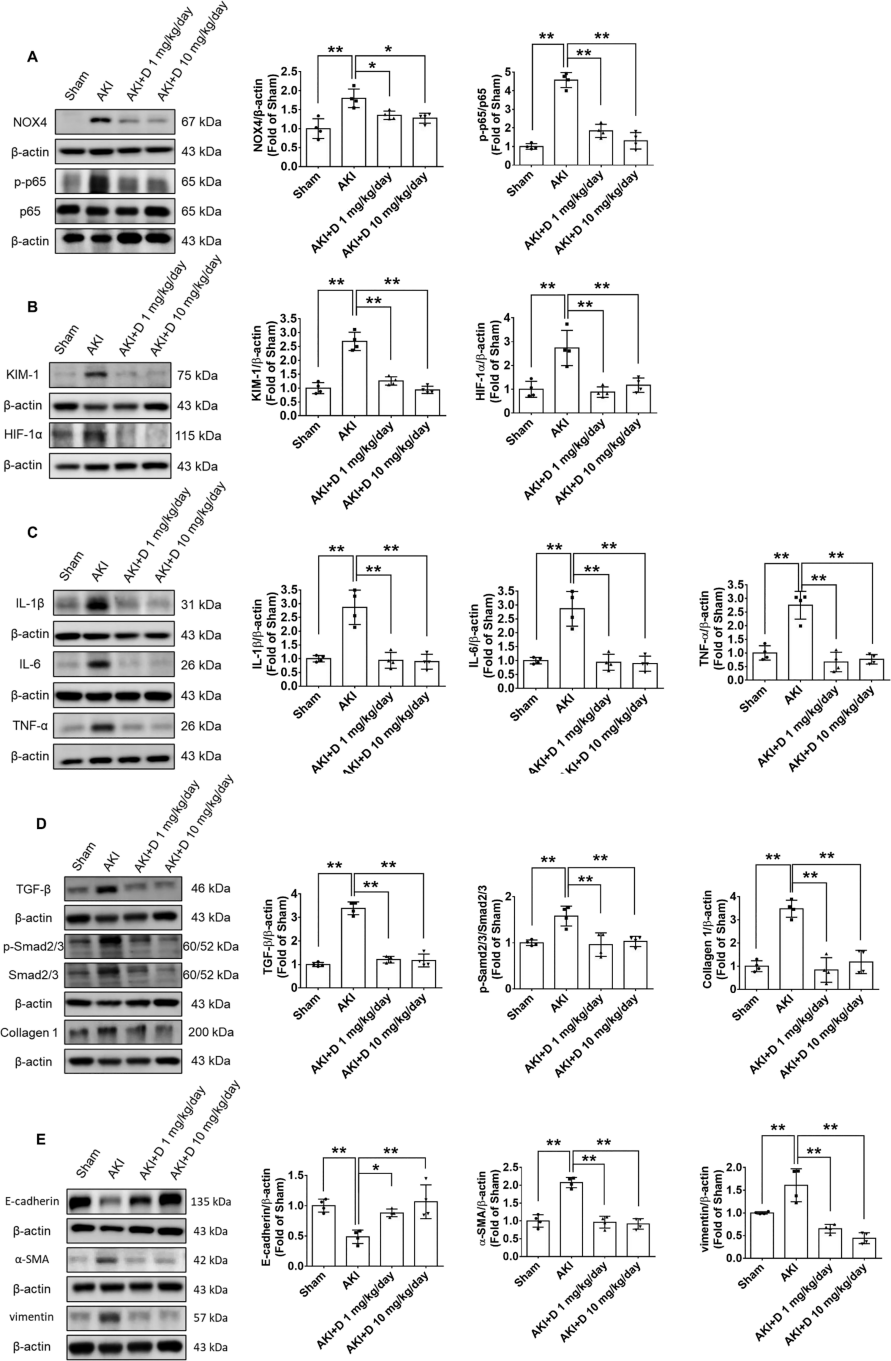
Diosgenin treatment reduced renal inflammation, fibrosis, and EMT factors in the chronic phase of AKI. Western blotting and statistical analyses of NOX4, phospho-p65 (*n* = 4; A), KIM-1, HIF-1α (*n* = 4; B), IL-1β, IL-6, TNF-α (*n* = 4; C), TGF-β, phospho-Smad2/3, Smad2/3, collagen 1 (*n* = 4; D), E-cadherin, α-SMA, and vimentin (*n* = 4; E) expression in kidney tissues 3 weeks after AKI. AKI, acute kidney injury; α-SMA, α-smooth muscle actin; D, diosgenin; EMT, epithelial–mesenchymal transition; HIF-1α, hypoxia-inducible factor-1α; IL-1β, interleukin-1β; IL-6, interleukin-6; NOX4, NADPH oxidase 4; KIM-1, kidney injury molecule-1; TGF-β, transforming growth factor-β; TNF-α, tumor necrosis factor-α. *P < 0.05, **P < 0.01.

The treatment with diosgenin for 3 weeks attenuated the upregulation of NOX4 and the phosphorylation of NF-κB subunit p65, which is regarded as a key factor associated with inflammation (Figure 3A). The administration of diosgenin also reduced the expression of tubular injury-related proteins, including KIM-1 and HIF-1α after 3 weeks of AKI in the kidney tissues of the AKI mice (Figure 3B). Furthermore, diosgenin-treated AKI mice led to a downregulation of inflammatory markers, including IL-1β, IL-6, and TNF-α, which were elevated due to AKI (Figure 3C). The diosgenin treatment also downregulated the renal fibrosis signaling pathways, such as TGF-β, phosphorylated Smad2/3, and collagen 1 levels after 3 weeks of AKI in the kidney tissues of AKI mice (Figure 3D). In addition, diosgenin regulated the expression of EMT-related proteins, such as E-cadherin, α-SMA, and vimentin, in the kidneys of the AKI mice (Figure 3E). Based on these finding, the administration of diosgenin in AKI mice improves both AKI and AKI-to-CKD progression by suppressing renal inflammation, fibrosis, and EMT signaling pathways.

### Diosgenin Downregulated the Hypoxia-Induced Oxidative Stress and the Expression of Inflammatory, Fibrotic, EMT, and Apoptotic Proteins in Human Renal Tubular Epithelial Cells

**Figure 4:**
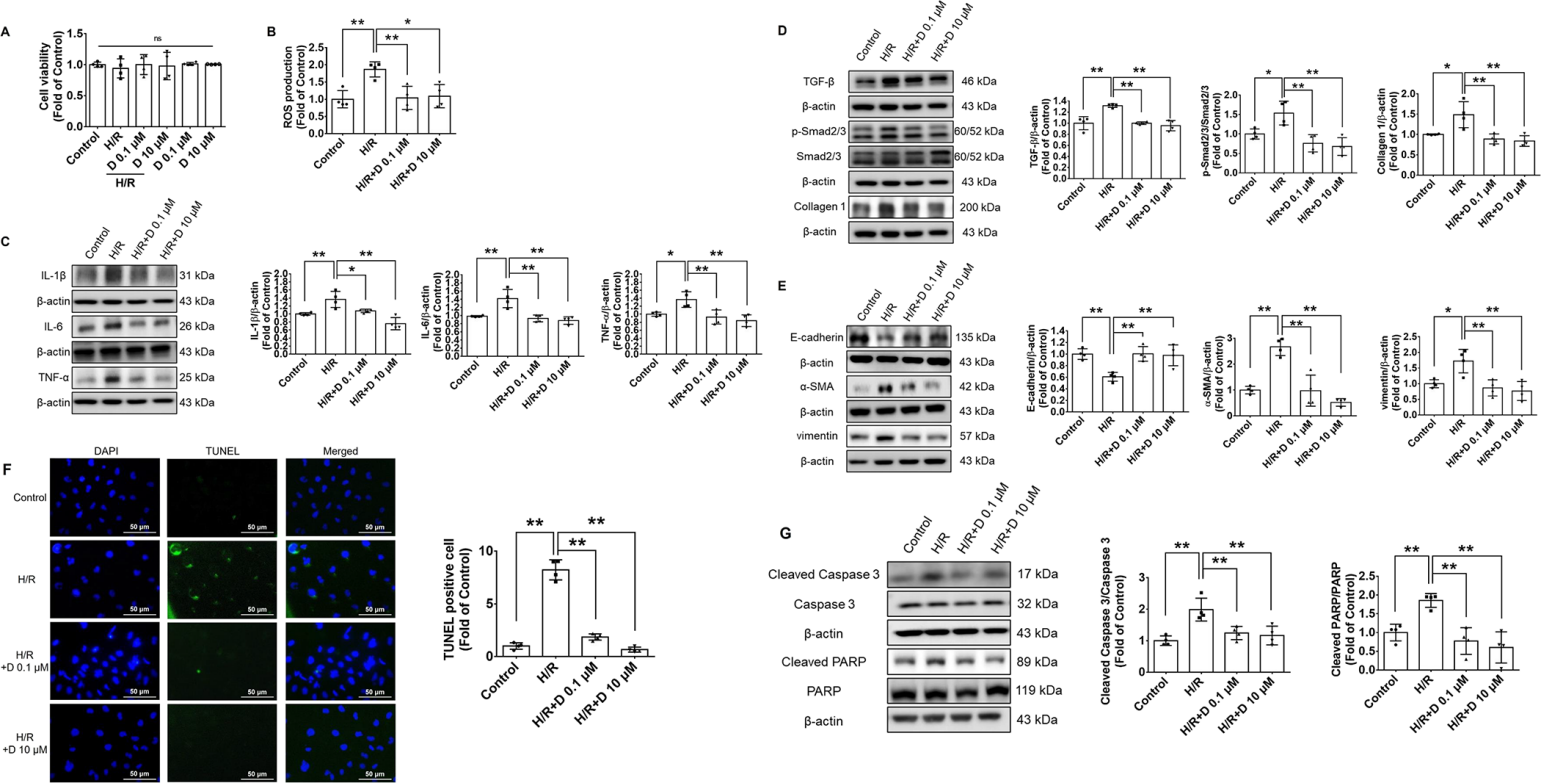
Diosgenin downregulated the hypoxia-induced oxidative stress, inflammation, fibrosis, and apoptosis in human renal proximal tubular epithelial cells. Cell viability of human renal tubular epithelial cells (*n* = 4; A). ROS production after hypoxia stimuli in human renal proximal tubular epithelial cells (*n* = 4; B). Western blotting and statistical analyses of IL-1β, IL-6, TNF-α (*n* = 4; C), TGF-β, phospho-Smad2/3, Smad2/3, collagen 1 (*n* = 4; D), E-cadherin, α-SMA, and vimentin (*n* = 4; E) expression in hypoxia-stimulated renal proximal tubular epithelial cells. Representative and quantitative analysis of TUNEL staining in hypoxia-stimulated renal proximal tubular epithelial cells (*n* = 4; F). Western blotting and statistical analyses of cleaved caspase 3 and cleaved PARP levels in hypoxia-induced renal proximal tubular epithelial cells (*n* = 4; G). α-SMA, α-smooth muscle actin; D, diosgenin; H/R, hypoxia/reperfusion; IL-1β, interleukin-1β; IL-6, interleukin-6; PARP, poly-ADP-ribose polymerase; ROS, reactive oxidative species; TGF-β, transforming growth factor-β; TNF-α, tumor necrosis factor-α. *P < 0.05, **P < 0.01.

The in vivo protective mechanism of diosgenin was further confirmed by using human renal tubular epithelial cells. Cell viability of human renal tubular epithelial cells was not affected by the treatment with diosgenin alone. While not affecting the cell viability (Figure 4A), diosgenin showed an antioxidative effect by attenuating the ROS generation (Figure 4B) and exerted an anti-inflammatory effect via reducing the expression of inflammatory proteins, including IL-1β, IL-6, and TNF-α in the hypoxia-stimulated renal tubular epithelial cells (Figure 4C). Additionally, diosgenin reversed the expression of fibrotic and EMT-related proteins, including TGF-β, phospho-Smad2/3, collagen 1, E-cadherin, α-SMA, and vimentin, which are highly associated with AKI-to-CKD progression (Figure 4D,E). The diosgenin treatments also prevented hypoxia-induced cell apoptosis and suppressed the expression of cell apoptosis-related proteins, including cleaved caspase 3 and cleaved PARP (Figure 4F,G). These results showed that diosgenin could protect human renal tubular epithelial cells from the damage of in vitro hypoxia injury by decreasing oxidative stress and the expression of inflammatory, fibrotic, EMT, and apoptotic proteins.

### Diosgenin Protected Renal Tubular Epithelial Cells from Hypoxia Injury through NOX4 Inhibition

**Figure 5:**
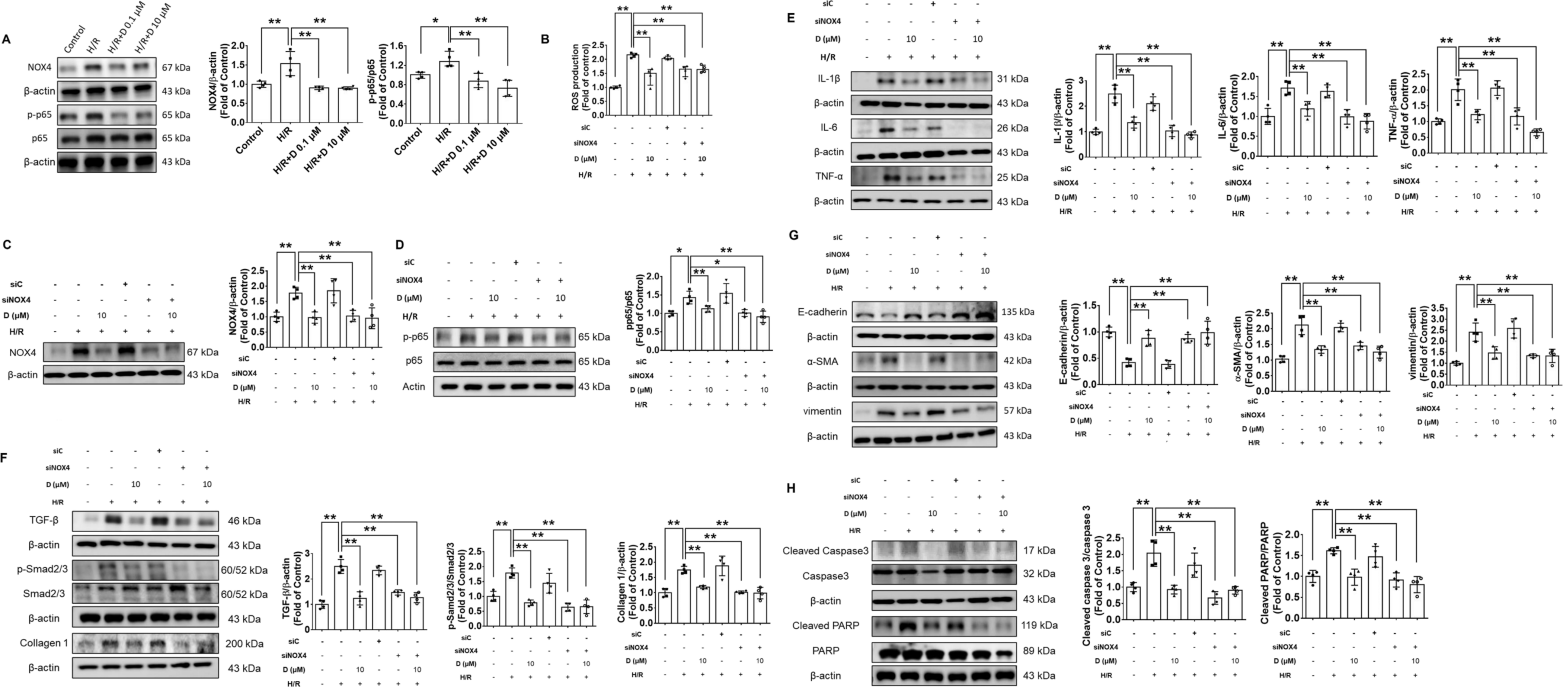
Diosgenin decreased the hypoxia-stimulated renal tubular epithelial cell damage via the NOX4/p65 signaling pathway. Western blotting and statistical analyses of NOX4 and phospho-p65 levels in hypoxia-stimulated renal proximal tubular epithelial cells (*n* = 4; A). ROS production in NOX4-knockdown renal tubular epithelial cells under hypoxia conditions (*n* = 4; B). Western blotting and statistical analyses of NOX4 and phospho-p65 levels after the administration of NOX4 siRNA in hypoxia-stimulated renal proximal tubular epithelial cells (*n* = 4; C, D). Western blotting and statistical analyses of IL-1β, IL-6, TNF-α (*n* = 4; E), TGF-β, phospho-Smad2/3, Smad2/3, collagen 1 (*n* = 4; F), E-cadherin, α-SMA, vimentin (*n* = 4; G), cleaved caspase3, and cleaved PARP (*n* = 4; H) levels after the administration of NOX4 siRNA in hypoxia-stimulated renal proximal tubular epithelial cells. α-SMA, α-smooth muscle actin; D, diosgenin; H/R, hypoxia/reperfusion; IL-1β, interleukin-1β; IL-6, interleukin-6; NOX4, NADPH oxidase 4; PARP, poly-ADP-ribose polymerase; ROS, reactive oxidative species; TGF-β, transforming growth factor-β; TNF-α, tumor necrosis factor-α. *P < 0.05, **P < 0.01.

The expression of NOX4 and phospho-p65 proteins was enhanced after the induction of hypoxia but reduced under the diosgenin treatments (Figure 5A). The siRNA-mediated knockdown of NOX4 suppressed hypoxia-induced ROS production. Notably, there was no significant difference in the reduction of ROS levels between the group treated with both diosgenin and NOX4 siRNA and the group treated with NOX4 siRNA alone, implying that the beneficial effect of diosgenin (i.e., the reduction in ROS levels) might be mainly dependent on the NOX4 signaling pathway (Figure 5B). The hypoxia-induced expression of NOX4 and phospho-p65 were markedly attenuated by the administration of NOX4 siRNA in the human renal tubular epithelial cells (Figure 5C,D). Moreover, the expression of inflammatory, fibrotic, and EMT-related proteins, such as IL-1β, IL-6, TNF-α, TGF-β, phospho-Smad2/3, collagen 1, E-cadherin, α-SMA, and vimentin, was also reversed by the administration of NOX4 siRNA (Figure 5E–G). The knockdown of NOX4 also alleviated the expression of cell apoptosis-related proteins, including cleaved caspase3 and cleaved PARP, in human renal tubular epithelial cells under hypoxic conditions (Figure 5H). Importantly, there was no significant difference in the expressions of phospho-p65, and inflammatory, fibrotic, EMT, and apoptosis-related proteins between the group treated with both diosgenin and NOX4 siRNA and the group treated with NOX4 siRNA alone ((Figure 5D–H). Accordingly, diosgenin may exert antioxidative, anti-inflammatory, antifibrotic, and antiapoptotic effects through modifying NOX4/p65 signaling pathways in human renal tubular epithelial cells under hypoxia conditions that mimic in vivo I/R.

## Discussion

**Figure 6:**
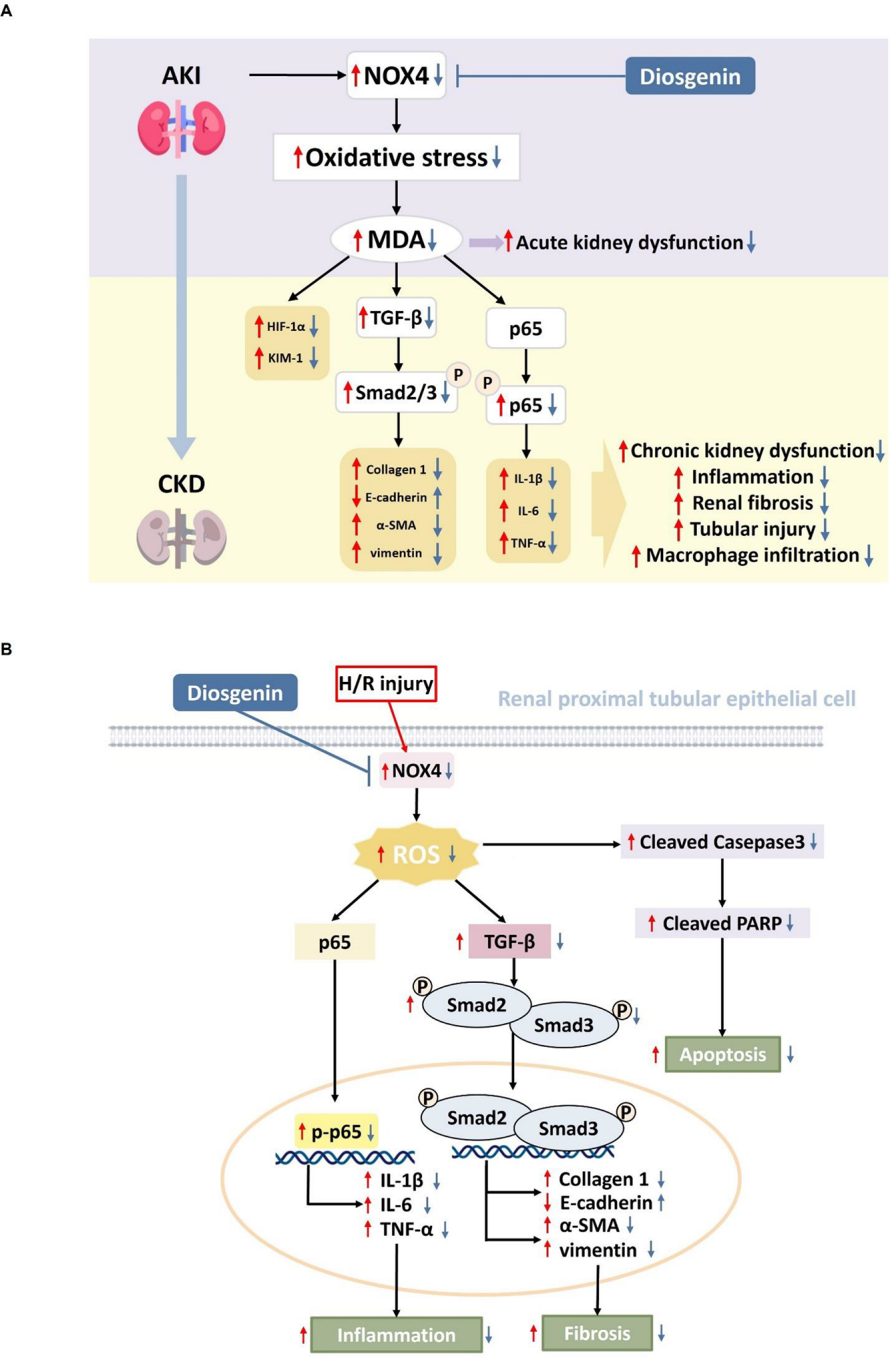
Diosgenin reduced kidney damages from AKI and prevents the progression of AKI to CKD. Diosgenin treatment not only protected kidney from I/R injury in both acute and chronic phases, but also inhibited the progression of AKI to CKD in vivo (A). Diosgenin downregulated hypoxia-stimulated cellular damage through the NOX4/p65 signaling pathway in human renal tubular epithelial cells (B). AKI, acute kidney injury; α-SMA, α-smooth muscle actin; CKD, chronic kidney disease; D, diosgenin; H/R, hypoxia/reperfusion; IL-1β, interleukin-1β; IL-6, interleukin-6; NOX4, NADPH oxidase 4; MDA, malondialdehyde; PARP, poly-ADP-ribose polymerase; ROS, reactive oxidative species; TGF-β, transforming growth factor-β; TNF-α, tumor necrosis factor-α.

In this study, we demonstrated the in vivo renoprotective effects of diosgenin in both I/R-induced AKI and the consequent development of CKD after AKI. Diosgenin exerted an antioxidative effect with reduced serum MDA levels in AKI mice. Diosgenin also improved renal function with reduced serum BUN and creatinine levels as well as UACR in acute and chronic phases of AKI. Importantly, the administration of diosgenin after the induction of AKI improved the renal damage by attenuating tubular injury, macrophage infiltration, and fibrosis in the kidney tissues of AKI mice. It also downregulated the expression of inflammatory, fibrotic, and EMT-related proteins, including IL-1β, IL-6, TNF-α, TGF-β, phospho-Smad2/3, collagen 1, α-SMA, and vimentin in mouse kidney tissues through 21 days after the induction of AKI. On the other hand, diosgenin protected human renal tubular epithelial cells from in vitro hypoxia injury that mimic in vivo I/R by decreasing oxidative stress and inflammatory, fibrotic, EMT, and apoptotic protein expression through the NOX4/p65 signaling pathway. Taken together, diosgenin presents renoprotective effects in I/R-induced AKI and the AKI progression to CKD by protecting renal proximal tubular epithelial cells through its effects probably through modifications of the NOX4/p65 signaling pathways. Our in vivo and in vitro findings support diosgenin as a potential protective strategy for the progression of ischemia-related AKI and consequent CKD (Figure 6).

AKI is closely related to the development of CKD, and AKI is the major factor accelerating the progression to CKD.^27^ The unrepaired damage caused by AKI may lead to renal fibrosis, which is a key factor in promoting the progression to CKD.^28^ I/R injury is one of the major causes of AKI, and limited oxygen uptake caused by AKI is a strong trigger for ROS production.^29^ NOX4 is highly expressed in the kidney under pathological conditions and plays a key role in I/R-induced ROS production in renal tubular epithelial cells.^11,12^ Given that NOX is the main source of ROS,^30^ the inhibition of the ROS produced by NOX may be a potential therapeutic approach for AKI and its consequent progression to CKD. A previous study revealed that diosgenin could suppress high glucose-induced ROS production by reducing NOX4 expression in renal tubular epithelial cells.^23^ Our current findings further suggested that diosgenin administration could not only decrease hypoxia-induced ROS production through its NOX4 inhibition in renal tubular epithelial cells in vitro but also reduce the renal damages during AKI and prevent consequent progression to CKD in vivo.

Previous papers also suggested the potential renoprotective effects of diosgenin by some other mechanisms in different types of renal injury. Diosgenin could protect against kidney injury induced by the food contaminant 3-MCPD by inhibiting endoplasmic reticulum stress and maintaining Ca^2+^ homeostasis and Bcl2 expression in human embryonic kidney cells.^31^ Diosgenin could also modulate autophagy through the AMPK–mTOR pathway and mitochondrial dynamics to protect against 3-MCPD-induced injury in human embryonic kidney cells.^22^ In addition, diosgenin could protect against aristolochic acid I-induced renal damage by upregulating Bcl2 and downregulating Bax and cleaved caspase-3,^32^ which is a key factor in apoptosis in AKI.^33^ Diosgenin could also attenuate calcium oxalate monohydrate-induced apoptosis and decrease oxidative stress in Madin-Darby canine kidney epithelial cells.^34^ Furthermore, diosgenin exhibited a protective effect in streptozotocin-induced diabetic nephropathy rats through the reduction of oxidative stress and inflammation.^35^ In addition, diosgenin was shown to prevent high glucose-induced kidney fibrosis by suppressing the EMT progression in renal tubular epithelial cells.^26^ On the other hand, diosgenin could not only suppress oxidative stress-induced inflammatory factors in high-fat diet mice^36^ but also inhibit the activation of NF-κB and expression of downstream proteins in Wistar rats fed an atherogenic diet.^37^ These observations imply that diosgenin may be a potential anti-inflammatory and antifibrotic reagent that could be used to treat various types of kidney diseases. In the current study, diosgenin was shown to maintain kidney function in both acute and chronic AKI phases and attenuate the AKI progression to CKD by decreasing tubular injury, macrophage infiltration, renal inflammation, and fibrosis in vivo. In the in vitro experiment, diosgenin downregulated hypoxia-induced oxidative stress and inflammatory, fibrotic, EMT, and apoptotic proteins in human renal tubular epithelial cells through the NOX4/p65 signaling pathway. The findings of this study are in line with previous data of the renoprotective effects of diosgenin in different in vitro and in vivo models and provide a novel rationale for the potential use of diosgenin for renoprotection in ischemia-induced AKI.

There are some limitations of our current study. First, although the protective effects of diosgenin on kidney function were shown in both in vitro and in vivo AKI models, there were no consistent dosage effects on renoprotection. Previous research has shown that diosgenin could mitigate cell death in human embryonic kidney cells exposed to 3-MCPD and inhibit certain enzymes at concentrations of 2, 6, and 8 μM, although without a clear dose-dependent pattern.^22^ Interestingly, another study reported that diosgenin could reduce high glucose-induced fibrosis in human renal tubular epithelial cells in a dose-dependent manner at concentrations of 0.1, 1, and 10 μM.^26^ In the present study, although not significantly, there seems to be a trend of the better effects of high dose (10 μM) rather than low dose (0.1 μM) of diosgenin on hypoxia stimulated cellular changes. Given that diosgenin did not affect cell viability at the currently selected concentrations, we preferred the high dose (10 μM) in combination with the siNOX4 to clarify the potential signaling pathways of diosgenin. Future experiments are required to justify the optimal doses of diosgenin in each individual in vitro and in vivo model before it could be considered for clinical experiments. Second, while I/R injury may be one of the leading causes of AKI,^6^ there are other potential causes of AKI, such as renal hypoperfusion, nephrotoxin exposure, and sepsis in clinical settings.^1,4,5^ Future studies may be needed to validate the potential renoprotective effect of diosgenin in each individual AKI model, such as cisplatin nephropathy, septic shock, and lipopolysaccharide-induced AKI. Third, while diosgenin alone treatment did not show negative effects on renal function in chronic renal failure rats in previous in vivo studies^38^ and on cell viability in human renal tubular epithelial cells in previous^39^ and current in vitro studies, the potential in vivo effects of diosgenin should be also addressed by alone treatment in the sham group of AKI for further comparison in this study. Fourth, given the potential significant impacts of diosgenin on ROS-NOX4 and NF-κB related mechanisms in other experimental models as indicated in the previous studies, the in vivo changes of protein expression related to the NOX4 signaling pathways were mainly evaluated for AKI and consequent CKD in the current study. Other redox-related or nonrelated mechanisms and signal pathways should be further investigated in future studies. Fifth, mainly Western blotting was used to investigate the protein expression and cell signaling in the current study. Given the potential complex effects of diosgenin, other readouts including the evaluation of mRNA and renal electronic microscopic changes and others may be required for further elucidation in future in vivo and in vitro studies. Finally, although in the current study diosgenin exerted its beneficial effects immediately, even at low doses during renal injury, the optimal dose and timing of clinical use should still be verified in future clinical trials.

In conclusion, our findings indicate that diosgenin may exhibit renoprotective properties against I/R-induced kidney injury by protecting renal proximal tubular epithelial cells through suppressing NOX4 activation and inhibiting ROS production to prevent consequent cell inflammation, fibrosis, and apoptosis. Given the lack of effective pharmacological interventions for AKI and AKI-to-CKD progression in the current clinical setting, future clinical trials may be worthy to validate if diosgenin treatment could be a potential protective strategy for ischemia-induced AKI and related CKD.

## Abbreviations

AKI: Acute kidney injury
BUN: Blood urea nitrogen
CKD: Chronic kidney disease
EMT: Epithelial–mesenchymal transition
H&E: Hematoxylin and eosin
HIF: Hypoxia-inducible factor
H/R: Hypoxia/reperfusion
IL: Interleukin
I/R: Ischemia/reperfusion
KIM-1: Kidney injury molecule-1
MDA: Malondialdehyde
NOX: NADPH oxidase
NF-κB: Nuclear factor kappa B
PAS: Periodic acid–Schiff
PARP: Poly-ADP-ribose polymerase
ROS: Reactive oxidative species
SMA: Smooth muscle actin
TGF-β: Transforming growth factor-β
TNF-α: Tumor necrosis factor-α
UACR: Urine albumin/creatinine ratio
