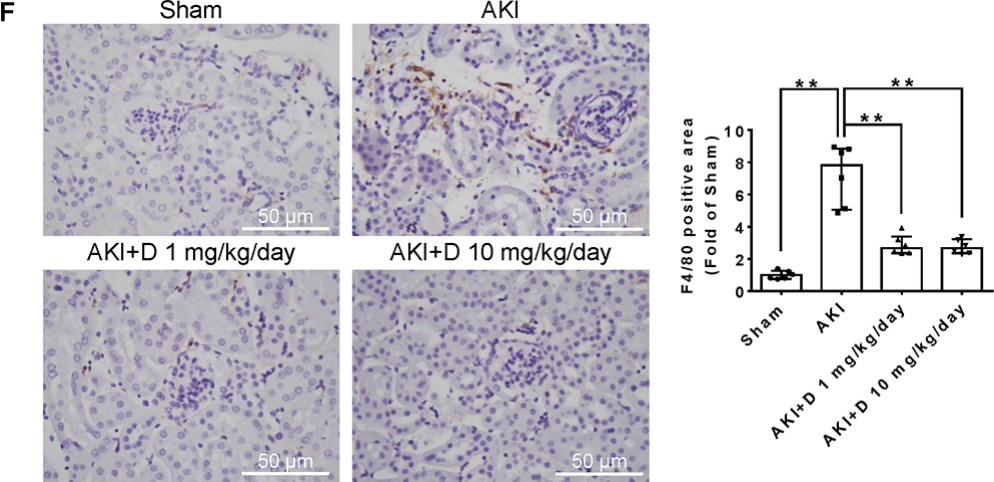# Correction to “Diosgenin Reduces Acute Kidney Injury and Ameliorates the Progression to Chronic Kidney Disease by Modifying the NOX4/p65 Signaling Pathways”

**DOI:** 10.1021/acs.jafc.4c09385

**Published:** 2024-10-19

**Authors:** Chih-Hung Chiang, Tien-Yun Lan, Jung-Hung Hsieh, Su-Chu Lin, Jaw-Wen Chen, Ting-Ting Chang

A higher-resolution version of Figure 2F is provided below.

**Figure 2:**